# Exploring palliative and end-of-life care perspectives and lived experiences among generations of African migrants residing outside the continent: A scoping review

**DOI:** 10.1017/S1478951525000070

**Published:** 2025-02-28

**Authors:** Salatiel Ngezi, Ashleigh E. Butler, Evelien Spelten

**Affiliations:** 1 La Trobe Rural Health School, La Trobe University; 2 School of Nursing and Midwifery, La Trobe University

**Keywords:** Palliative and end-of-life care, African migrants, perceptions, preferences, experiences

## Abstract

**Objectives:**

This review aimed to chart existing literature and identify gaps in the evidence base concerning palliative and end-of-life care perspectives and experiences among different generations of African migrants residing outside the continent.

**Methods:**

This review adhered to a predefined protocol, utilizing the Arksey and O’Malley 5-stage framework, as refined by Danielle Levac and colleagues. A systematic search of 5 bibliographic databases (from inception to December 2022) yielded 79 published studies. After title, abstract, and full-text screening using Covidence®, 7 studies met the inclusion criteria. Data extraction was guided by a conceptual framework tailored to the research topic and questions, with results presented in the narrative form.

**Results:**

Cultural and religious beliefs and practices significantly shaped African migrants’ perspectives on end-of-life care. A nuanced boundary between palliative and curative care emerged, with the former often stigmatized and stereotypically associated with death and dying. Common barriers to accessing end-of-life care included limited awareness, low literacy, and perceived inadequacy of culturally sensitive care, resulting in disparities in both access and outcomes. Additionally, reluctance to discuss death and dying, along with mistrust of Western healthcare systems, constituted significant obstacles. The studies underscored the necessity of enhancing provider–patient communication by engaging with migrants to raise awareness of services and fostering inclusive healthcare environments for improved care outcomes.

**Significance of results:**

Existing research on racial and ethnic disparities underscores the unequal quality and outcomes of end-of-life care across various racial groups. However, there is still insufficient understanding of these diverse end-of-life care needs, particularly in host countries. Bridging this knowledge gap is crucial for reducing health disparities and enhancing the delivery of culturally sensitive care within Western healthcare systems.

## Introduction

International migration has historically influenced population dynamics and remains a major concern for policymakers globally (Ozturk 2022; Pooley 2017; Rampazzo et al. 2023). According to a recent United Nations report, the global population of international migrants in 2020 was approximately 281 million (McAuliffe and Triandafyllidou 2022). Among these migrants, around 40.6 million individuals were of African origin (Nyaoro 2023). Notably, the number of African migrants has more than doubled since 1990, making it one of the most rapidly growing subpopulations migrating to Western countries (Hugo 2010; International Organization for Migration 2024; Venters and Gany 2011).

Amidst a global migration phenomenon involving over a billion people (World Health Organisation 2022), some migrant-receiving nations face challenges to their public health policies and programs due to increased cultural diversity and shifts in population demographics (Gushulak et al. 2009; Renzaho 2023). Consequently, there are concerns about meeting the diverse healthcare needs of emerging multicultural and multiethnic communities (Davies 2016; El Alaoui-Faris 2022). Although not equally distributed across all migrant groups, the research identifies that the racial/ethnic cohort experiences a higher prevalence of factors associated with vulnerability (Gushulak et al. 2009). Race, ethnicity, pre- and post-migration lifestyles, behaviors, and genetic predispositions to certain illnesses often link to health disparities among migrants and other racial and ethnic minorities (Belahsen 2022; Montesi et al. 2016; Renzaho et al. 2016). Despite the healthy migrant hypothesis, which suggests that migrants from certain countries experience lower disease rates than host populations (El Alaoui-Faris 2022), acculturation causes this advantage to wane over time. As migrants adapt and adjust to new environments, research indicates evidence of post-migration disadvantage and a shift in their health status to gradually align with that of native populations (Idemudia 2020; Montgomery et al. 2016; Ozturk 2022).

Migrating between different health environments affects the longer-term epidemiology of diseases and health outcomes at migrant destinations (Greenaway and Castelli 2019; Gushulak et al. 2009). In high-income countries, migration also significantly contributes to an aging demographic. According to the United Nations (UN DESA 2020), in 2020, more than 1 in 10 individuals aged 65 years or older were international migrants. Recent research indicates that older migrants have higher rates of hospitalizations, morbidity, and mortality related to age-related chronic and incurable conditions compared to the host society (El Alaoui-Faris 2022; Herring and Ezeofor 2023; Reis et al. 2022). As global population mobility trends continue, by 2056, this vulnerable, older population is projected to become much larger and more diverse and may require palliative care due to chronic and life-limiting illnesses (Adsersen et al. 2023; Kruja 2022; Spelten et al. 2021; Verne 2022; Wilson et al. 2020). Migrant-receiving countries must enhance their capacity and preparedness to incorporate the healthcare needs and preferences of migrants into their healthcare systems (Belahsen 2022). Additionally, a deeper understanding is needed regarding how specialized services, such as palliative care, may or may not translate effectively across diverse contexts (Samuels and Lemos Dekker 2023).

Palliative care has evolved into a distinct and essential healthcare specialty since the late 20th century. Rather than solely focusing on end-of-life care, palliative care prioritizes enhancing the quality of life for patients and caregivers, regardless of the illness or stage (Ditillo 2002; Grisold and Grisold 2022). Despite growing recognition, the global provision of palliative care remains inadequate. Approximately 20 million people require end-of-life palliative care annually, yet only 12–14% receive it (Verne 2022; World Health Organisation 2020). Furthermore, only about 30 countries have comprehensive policies, protocols, and specialized capacity to provide palliative care (Clark et al. 2020; Hawley 2017; Sleeman et al. 2019).

Research suggests that migrant populations may have limited awareness and knowledge of Western palliative care models, potentially exacerbating disparities in access to care (Adsersen et al. 2023; Carlsson and Hjelm 2021; Grisold and Grisold 2022). This issue is particularly concerning given the projected 87% increase in the burden of health-related suffering amenable to end-of-life interventions by 2060 (Clark et al. 2020; Sleeman et al. 2019).

Despite growing attention to migrants’ health, disparities in health outcomes persist, adversely affecting their quality of care. Addressing health inequities requires a multifaceted approach, including raising awareness among migrant communities and enhancing healthcare providers’ cultural competencies regarding palliative care access and utilization (Fares et al. 2023; Gerber et al. 2020; Möller et al. 2021; Tan et al. 2021). Health research must collect customized, racial, and ethnic data to inform policy interventions and service delivery, addressing the needs of migrant populations (Renzaho 2023; Torensma et al. 2021; Venters and Gany 2011). By identifying gaps in the current literature, this review aims to highlight research deficiencies and offer an insight for future investigations into the end-of-life care perspectives of African migrants. Definitions of different terms used in this review are listed in Table 1 for clarity.

**Table 1.** Key phrases, concepts, and terms used in this review

African migrant	In the context of this review, this term refers to individuals who self-identify as having full or partial ancestry from the African continent, either by birth or descent, and who have emigrated outside of Africa, making the destination country their new usual place of residence (Fouche et al. 2021)
Palliative care	Palliative care is an approach designed to enhance the quality of life for patients and their families dealing with life-threatening illnesses. It focuses on alleviating pain and symptoms, as well as providing spiritual and psychosocial support (World Health Organisation 2020)
End-of-life care	End-of-life care includes physical, spiritual, and psychosocial support for patients expected to die within 12 months, including those with life-threatening acute conditions. It also extends to support for families and carers, as well as post-death care (Australian Institute of Health and Welfare 2016)
Cultural safety	First introduced in New Zealand’s health education in the 1990s, this concept requires healthcare systems to be accountable for providing culturally safe care as defined by patients and their communities. This involves continuous self-reflection and self-awareness (Curtis et al. 2019)
Ethnicity	This term refers to the differentiation and identification of people based on group membership and shared belief systems, such as religion, traditions, customs, language, heritage, or ancestry (Renzaho 2023)
Culturally Competency	This is a broad concept that includes values, behaviors, attitudes, knowledge, and skills enabling professionals to provide patient care that is respectful and inclusive of diverse cultural backgrounds, aiming to reduce ethnic health disparities (Evans et al. 2012)
Culturally and linguistically diverse (CALD)	A culturally diverse community is characterized by a wide range of attributes, including diverse languages, ethnic backgrounds, nationalities, dress, traditions, food, societal structures, art, and religions (Department of Health and Aged Care 2019)

## Methods

A scoping review was conducted from February 2023 to August 2024, guided by a preregistered protocol (DOI: 10.17605/OSF.IO/83WSB). This review followed the 5-stage framework for scoping reviews developed by Arksey and O’Malley (2005) and later refined by Levac et al. (2010).

Arksey and O’Malley’s (2005) 5-stage framework involves (1) identifying the research question, (2) locating relevant studies, (3) selecting studies, (4) charting the data, and (5) collating, summarizing, and reporting the results. This framework establishes a foundational process for scoping reviews. Levac et al. (2010) refined this framework by emphasizing the need to clarify and link the study’s purpose with the research questions and by using an iterative team approach for literature search, study selection, and data extraction to improve methodological rigor. These refinements aim to enhance the transparency and comprehensiveness of scoping reviews.

### Stage 1: identifying the review questions

This stage focused on the body of evidence in the included studies, to answer the following research questions:

1. What is known about palliative and end-of-life care experiences among African migrant communities living outside of Africa?
2. What specific experiences, challenges, or facilitators are reported by African migrant communities regarding palliative and end-of-life care services?
3. How do existing policies and procedures impact the adoption and provision of palliative and end-of-life care for African migrant communities?

### Stage 2: identifying relevant studies

A comprehensive search strategy was developed iteratively in collaboration with a research librarian (Appendix B). We applied the PCC mnemonic (Population, Concept, and Context) to create a search algorithm and inclusion/exclusion criteria (Appendix A), as recommended by Pollock et al. (2023).

We conducted a thorough literature search across major health science databases, including PsycINFO, Medline, Embase, PubMed, and CINAHL. The search was limited to peer-reviewed studies in English, published from each database’s inception until December 2022. Eligible studies included full-text qualitative or mixed-methods research. To ensure a comprehensive search, we also examined reference lists from relevant journals, conducting grey literature searches for policy documents and reports from Australian government websites and palliative care organizations such as Palliative Care Australia, Australian Institute of Health and Welfare, and Australian Healthcare and Hospitals Association, and searched online repositories, Google, and Google Scholar.

### Stage 3: study selection

We systematically compiled and uploaded the identified studies to Covidence® software (Covidence.org, n.d.). Using Covidence, we created a Preferred Reporting Items for Systematic Reviews and Meta-analyses (PRISMA) flowchart (Table 1) to illustrate the study selection process. Two reviewers independently screened titles and abstracts, guided by the inclusion and exclusion criteria. We resolved conflicts arising during this process through consensus or third-reviewer arbitration, providing a clear rationale for excluded studies.

### Stage 4: charting the data

The first author designed a data extraction form (Table 2) to guide the sifting, charting, and sorting of data from eligible studies. The review team developed a conceptual framework aligned with the research topic and questions to guide the extraction of descriptive data, as suggested by Levac et al. (2010). After feedback and trials, the final form was adopted, capturing author details, publication country and year, study aims, methodology, participant characteristics, outcomes, and key results.

**Table 2.** Characteristics of the extracted studies

First author and year Country of origin	Population of focus	Study aims	Phenomenon of interest	Design	**Important results** **Recurring themes specific to review questions**
Ben-Arye et al. (2018) Germany	Clinicians, medical educators, and research participants in a workshop, focusing on Middle Eastern and northern African countries’ refugees in Europe *N* = Not specified	Aims… to address the challenges faced by European health professionals when dealing with the recent wave of migration from Middle Eastern countries	Traditional health belief models of refugees	Qualitative research	This German study emphasizes the importance of healthcare professionals understanding diverse health belief systems and enhancing communication, particularly in palliative and cancer care. It recommends incorporating traditional medicine and advanced communication skills into training programs to deliver culturally sensitive care, especially for refugee and Muslim communities
Coe (2020) United States	African migrant home care workers and their patients’ families, living in the United States *N* = 87	Aims… to explore the role of home health workers in facilitating the process of dying at home	Mediation of death by home health workers	Qualitative research	In the United States, this research highlights the essential role of African migrant home care workers in ensuring a peaceful end-of-life transition for patients. These workers act as crucial allies for patients and surrogates, fostering a serene transition. Their professional intimacy forms a significant, though temporary, bond with the patient’s family, underscoring the importance of their contributions during the patient’s final journey
de Graaff et al. (2012) Netherlands	Turkish and Moroccan migrants living in the Netherlands *N* = 83	Aims… to explore how communication and decision-making in palliative care among Turkish and Moroccan patients is influenced by unique styles of care management …	Communication and decision-making in palliative care	Qualitative research	Conducted in the Netherlands, this study examined communication and decision-making challenges in palliative care among Turkish and Moroccan migrants. It highlighted cultural conflicts and the necessity for healthcare professionals to better understand these communities. Furthermore, ineffective communication among care providers often complicates decision-making processes, hindering migrant patients’ access to curative options
Hiruy and Mwanri (2014) Australia	Ethiopian, African male and African migrants living in Australia *N* = Not specified	Aims… to provide some cultural insights into how some African communities perceive the provision of healthcare in general and end-of-life care …	Palliative and hospice care	Qualitative, phenomenological case study	This Australian study highlights the necessity of incorporating religious and cultural considerations into end-of-life care for African communities. It advocates for the integration of these considerations into healthcare practices and calls for the development of ethical frameworks that balance Afro-communitarian principles with individual rights, ensuring universally relevant and respectful care for all patients
Maddalena et al. (2010) Canada	Decedents and primary caregivers in 3 African Canadian families *N* = 7	Aims… to examine the meanings that African Canadians ascribe to their cancer and end-of-life experiences	Cancer care and end-of-life care	Case study methodology using in-depth interviews	In Canada, this study underscores the preference for home-based end-of-life care among African Canadians and advocates for culturally competent health services. It emphasizes the need for informed policy-making and professional education to align with these preferences, recommending further exploration of cultural aspects of care within the community
Nyashanu et al. (2020) United Kingdom	African migrants from Sub-Sahara African (BSSA) countries, living in the United Kingdom *N* = 180	Aims… to explore perceptions and attitude of BSSA toward residential care from a potential user perspective	Residential care for elderly African migrants	Qualitative explorative research	This UK study found significant challenges faced by elderly African migrants in residential care, underscoring the necessity for culturally sensitive approaches. These challenges encompass a sense of confinement, lack of ownership, non-provision of culturally appropriate food, inadequate personal care, absence of culturally oriented end-of-life care, and stigma related to neglect. Consequently, these factors deter BSSA individuals from choosing residential care in the UK. The study advocates for culturally appropriate services and enhanced staff cultural competency to foster inclusivity and well-being among elderly BSSA migrants
Sneesby et al. (2011) Australia	Sudanese, African migrants living in Australia (volunteer participants aged 18–53 years, including 10 women) *N* = 15	Aims… to obtain information to support Palliative Care healthcare workers to meet the needs of the Sudanese population in death, dying, and bereavement	Palliative care	Qualitative interpretive research	This Australian study examines the palliative care needs of Sudanese migrants, emphasizing the significant role of spiritual and religious leaders in end-of-life decisions. It underscores the necessity of personalized palliative care consultations that honor the cultural diversity within Sudanese Australian communities. The research advocates for a nuanced understanding of everyone’s ethnic and religious background to deliver culturally congruent end-of-life care, thereby advancing palliative care practices and policies

### Stage 5: collating, summarizing, and reporting results

We used a narrative approach to collate and summarize findings that met our eligibility criteria, providing an overview of the included literature. Data from the 4 main concepts were summarized, compared, and contrasted to offer a comprehensive overview. The findings were reported narratively and discussed in relation to the review questions and objectives.

## Results

**Fig. 1.**
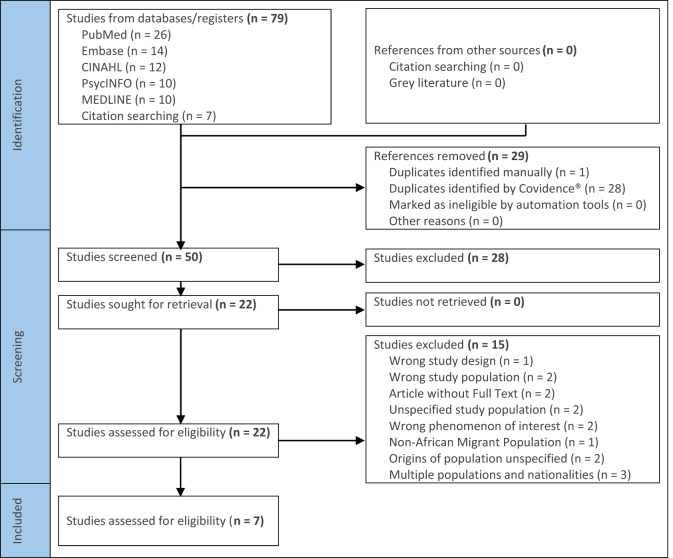
PRISMA-ScR flowchart of study selection process.

The article screening process is outlined in the PRISMA flowchart (Figure 1). Initially, 79 studies were identified. After removing duplicates and exclusions, 7 studies met the inclusion criteria, comprising 6 qualitative studies and one mixed-method case study.

### Study characteristics

This review includes 6 studies (2010–2020) conducted in Australia (*n* = 2), Canada, Germany, Netherlands, United Kingdom, and United States (*n* = 1 each). These studies employed mixed-methods approaches to investigate palliative and end-of-life care for African migrant communities, utilizing data from semi-structured interviews, focus groups, case studies, questionnaires, and observations (Ben-Arye et al. 2018; de Graaff et al. 2012; Hiruy and Mwanri 2014; Maddalena et al. 2010; Nyashanu et al. 2020; Sneesby et al. 2011).

### Participant characteristics

The included studies referred to participants using various terms, including “Black,” “immigrants,” “migrants,” “refugees,” “ethnic minorities,” and “African.” While 2 studies specified participants’ origins as Sudanese (Sneesby et al. 2011) and Turkish/Moroccan (de Graaff et al. 2012), the remaining 5 studies broadly identified Africa as the participants’ origin. Participants held diverse roles within community-based palliative and end-of-life care contexts, including patients, family members, caregivers, recipients of aged care services, and home health workers. Some participants also held roles as religious or spiritual leaders, neighbors, friends, relatives, community elders, leaders, or members. A summary of the extracted data is presented in Table 2.

The review findings are organized under 4 main concepts: perceptions, preferences, and experiences of care at end-of-life; challenges to engagement with palliative care; facilitators for engagement with palliative care; and policy and procedure impact on uptake and delivery of palliative care.

#### Concept 1: perceptions, preferences, and experiences of care at end-of-life

Traditional and spiritual beliefs significantly shaped African migrants’ perspectives on end-of-life care. Spiritual and religious leaders, including priests, pastors, sheikhs, and traditional healers, were pivotal in guiding decisions about end-of-life care for patients and families (Hiruy and Mwanri 2014; Maddalena et al. 2010; Sneesby et al. 2011). Additionally, paid caregivers incorporated spirituality into their practices to help patients strengthen their relationship with God in the final stages of life (Coe 2020).

Collectivist values and shared community identities strongly influenced attitudes toward illness management and interactions with healthcare systems (Hiruy and Mwanri 2014; Maddalena et al. 2010). Family and community involvement in decision-making was preferred over individual autonomy, contrasting with Western ideals of maintaining individual autonomy (de Graaff et al. 2012; Hiruy and Mwanri 2014; Sneesby et al. 2011). However, younger Sudanese migrants showed a shift toward more individualistic preferences, favoring direct communication with healthcare professionals (Sneesby et al. 2011).

Family-based caregiving at home was preferred over hospital-based care, with home considered the ideal setting for end-of-life care (Hiruy and Mwanri 2014; Maddalena et al. 2010). This preference reflected a cultural expectation among African migrant communities to care for sick relatives at home rather than utilizing acute or sub-acute care facilities (Hiruy and Mwanri 2014; Maddalena et al. 2010; Sneesby et al. 2011).

Sneesby et al. (2011) found that Sudanese participants viewed caring for older family members as both a privilege and a blessing. Similarly, Coe (2020) discovered that home healthcare workers drew upon their experiences caring for dying relatives to create meaningful end-of-life experiences for their patients. However, this preference was not universal. de Graaff et al. (2012) noted that some patients, particularly Turkish and Moroccan migrants, preferred hospital-based care to alleviate caregiver burden. Furthermore, research has shown that African migrants lacking extended family support often opt for institutionalized end-of-life care (Hiruy and Mwanri 2014; Nyashanu et al. 2020).

#### Concept 2: challenges to engagement with palliative care

African migrants faced challenges in engaging with healthcare services, including palliative care, in their host countries compared their countries of origin. Hiruy and Mwanri (2014) noted that navigating Western healthcare systems and limited awareness of palliative care services were key factors. For the Ethiopian migrant in their case study, palliative care services were either unknown or recently established in their home country. Similarly, Sneesby et al. (2011) found that Sudanese community members lacked awareness and experience of palliative care before emigrating.

Perceptions of Western medicine among African migrants were complex. Sudanese migrants viewed Western medicine as highly sophisticated and capable of curing various ailments (Sneesby et al. 2011). In other studies, traditional medical practices, including home remedies and folk medicine, often supplemented conventional medicine for managing terminal illness (Ben-Arye et al. 2018; Maddalena et al. 2010; Sneesby et al. 2011). The belief in traditional remedies blurred the distinction between palliative and curative care. Trust in Western medicine was often linked to the pursuit of curative and life-saving treatments (de Graaff et al. 2012; Maddalena et al. 2010; Sneesby et al. 2011).

Skepticism about adopting Western models of palliative and end-of-life care was common. Strong beliefs in the sanctity of life led some migrants to prioritize prayer and other religious or spiritual rituals over accepting the terminal nature of their disease (Hiruy and Mwanri 2014; Maddalena et al. 2010). Cultural norms and traditional practices, such as prayer and spiritual support, played a significant role in how migrants confronted mortality (Coe 2020; Hiruy and Mwanri 2014; Sneesby et al. 2011).

Beliefs and attitudes toward end-of-life care varied significantly, impacting perceptions of terminal illness, death, and dying. In 6 of the included studies, practitioners in Western countries faced challenges due to culturally and religiously constructed differences in healthcare beliefs and ethnic-cultural views when engaging with migrant patients (Ben-Arye et al. 2018; Coe 2020; de Graaff et al. 2012; Hiruy and Mwanri 2014; Maddalena et al. 2010; Nyashanu et al. 2020; Sneesby et al. 2011). These studies suggested that understanding these variations is crucial for providing care to people from diverse cultural backgrounds.

#### Concept 3: facilitators for engagement with palliative care

Effective communication, cultural sensitivity, and the promotion of cultural competency were common facilitators identified in the studies for engagement with palliative care services. Specifically, effective communication played a pivotal role in encouraging active participation in open and honest health and palliative care discussions (Ben-Arye et al. 2018; Coe 2020; de Graaff et al. 2012; Hiruy and Mwanri 2014; Maddalena et al. 2010; Nyashanu et al. 2020; Sneesby et al. 2011). Health professionals were encouraged to acknowledge and respect diverse cultural values and beliefs, giving equitable voices to individuals and group members within migrant communities (de Graaff et al. 2012; Hiruy and Mwanri 2014; Sneesby et al. 2011). Additionally, shared decision-making, especially involving families or communities, was highly valued (Ben-Arye et al. 2018; de Graaff et al. 2012; Hiruy and Mwanri 2014; Maddalena et al. 2010).

Bridging language and cultural gaps was essential for enhancing engagement with diverse patients (Ben-Arye et al. 2018; de Graaff et al. 2012; Hiruy and Mwanri 2014; Sneesby et al. 2011). These studies advocated for the active participation of both service providers and patients in joint decision-making processes to tailor end-of-life care. Training and professional development that incorporated cultural competency and inclusivity were crucial for effectively serving migrant communities (Ben-Arye et al. 2018; de Graaff et al. 2012; Hiruy and Mwanri 2014; Nyashanu et al. 2020). Additionally, insights from workshops recommended educational initiatives that consider transcultural health beliefs within Western healthcare models to improve engagement and participation (Ben-Arye et al. 2018). Recognizing and respecting the role of supportive family and community networks, as well as religious and spiritual beliefs, significantly influenced end-of-life care decision-making among individuals of African descent (Ben-Arye et al. 2018; Hiruy and Mwanri 2014; Maddalena et al. 2010).

#### Concept 4: policy and procedure, impact on uptake and delivery of palliative care

Disparities in accessing culturally appropriate care were highlighted, emphasizing the need for policymakers, healthcare providers, and scholars to integrate African cultural and ethical considerations into palliative care frameworks (Ben-Arye et al. 2018; Coe 2020; de Graaff et al. 2012; Hiruy and Mwanri 2014; Maddalena et al. 2010; Nyashanu et al. 2020; Sneesby et al. 2011). The reviewed studies suggested the importance of developing social and cultural competency tailored to migrant populations. Additionally, clinicians and policymakers were urged to address health challenges faced by migrants through accurate assessments and the creation of culturally safe spaces (Coe 2020; de Graaff et al. 2012). These safe spaces play an integral role in maintaining the customs and rituals of African migrants during the end-of-life phase (Maddalena et al. 2010).

## Discussion

The review examined the end-of-life care perceptions, preferences, and experiences of African migrants, highlighting the importance of cultural and religious beliefs in influencing their care choices. African migrants show a strong preference for home-based end-of-life care, influenced by cultural and religious beliefs, with families and communities playing a crucial role in supporting terminally ill relatives at home. However, language barriers, limited health literacy, and unfamiliarity with Western healthcare models hinder engagement with end-of-life care services. Furthermore, disparities in access to palliative and end-of-life care are associated with differences in ethnic-cultural backgrounds. To address these disparities, healthcare providers’ cultural competency and understanding of diverse needs are critical in bridging gaps in cultural understanding. Ultimately, policy strategies, such as targeted education campaigns and culturally sensitive care guidelines, are necessary to address healthcare disparities and provide equitable end-of-life care.

## End-of-life care: preferences and experiences

African migrants show a strong preference for home-based end-of-life care, influenced by cultural and religious beliefs, values, and familial responsibilities. Families and communities play a crucial role in supporting terminally ill relatives at home rather than relying on in-patient care (de Graaff et al. 2012; Hiruy and Mwanri 2014; Maddalena et al. 2010; Sneesby et al. 2011). Home-based care is also a transferable skill used by migrant Home Health Workers managing patients’ terminal phases within private homes (Coe 2020). The home environment offers emotional solace during the final days, providing comfort and familiarity often absent in institutional settings. It also allows for the practice of cultural and religious rites integral to the end-of-life phase. However, despite the privacy and independence of home-based care, the lack of external clinical expertise and support remains a challenge, leading most terminally ill patients to pass away in institutional settings (Donovan et al. 2011). The reviewed studies similarly observed that inadequate clinical capabilities within African migrants’ home-based support networks hinder handling complex medical requirements encountered during the end-of-life stage (de Graaff et al. 2012; Hiruy and Mwanri 2014; Sneesby et al. 2011). Addressing these challenges may involve promoting community and home-based hospice programs for African migrant communities, supervised by specialized, multidisciplinary palliative care teams (Cottrell and Duggleby 2016). Innovative technologies, such as telehealth platforms, may facilitate remote consultations and specialized clinical care from patients’ homes (Gordon et al. 2022; Steindal et al. 2020). However, despite these advancements, gaps persist in research on the spiritual, cultural, and ethical-legal dimensions of care, and the impact of home-based care on end-of-life outcomes (Chen et al. 2022; Shepperd et al. 2021; Sinclair et al. 2020).

## Barriers to palliative care engagement

The review highlighted communication as a significant barrier to effective healthcare access for African migrants, especially regarding discussions about death and dying. Four studies revealed a reluctance among African migrants to openly discuss, plan, and prepare for end-of-life matters, often considering these topics taboo and sensitive (de Graaff et al. 2012; Maddalena et al. 2010; Nyashanu et al. 2020; Sneesby et al. 2011). Language barriers and varying health literacy levels compound this reluctance, leading to delays in care and mistrust of Western healthcare practices. In palliative and end-of-life situations, inadequate and ineffective communication between patients and physicians contributes to delayed prognosis assessment and end-of-life care discussions, often occurring when patients are already nearing the end of life (Lowey 2015).

Research has consistently found that language differences, limited health literacy, and unfamiliarity with Western healthcare models hinder engagement with end-of-life care services among minority ethnic groups (Barwise et al. 2018; Gerber et al. 2020; Shabnam et al. 2022). Migrants with lower health literacy and limited English proficiency may develop misconceptions about palliative care. These factors create communication challenges with healthcare teams, disadvantaging migrants during care-seeking and treatment (Cappa and Canevelli 2022; Sze et al. 2015). Practitioners may hesitate to discuss end-of-life care due to concerns about effective communication and patient understanding, inhibiting open discussions in palliative and end-of-life contexts. Addressing these issues is crucial to ensure equitable and sensitive healthcare delivery in multicultural settings.

## Enablers of palliative care engagement

The review found that disparities in access to palliative and end-of-life care for CALD groups were associated with differences in ethnic-cultural backgrounds (de Graaff et al. 2012; Hiruy and Mwanri 2014; Maddalena et al. 2010; Sneesby et al. 2011). This aligns with WHO observations that migrant status is often linked to impaired health and poorer access to health services (World Health Organisation 2010).

In their study of African Canadians in Nova Scotia, Maddalena et al. (2010) highlighted how racism, marginalization, and historical exclusion by healthcare institutions impact African and other minority patients, contributing to disparities in accessing culturally appropriate palliative and end-of-life care. Discrimination and disadvantage manifest in various forms, such as unequal treatment, lack of cultural competency among providers, and systemic biases affecting the quality of care for socially defined groups (Krieger 2001). These challenges are common among other minority religious, ethnic, and racial groups disproportionately affected by social and health inequalities (Gerber et al. 2020; McAuliffe and Triandafyllidou 2022; Omenka et al. 2020; Renzaho 2023; Spelten et al. 2021). Recent studies have highlighted significant disparities in healthcare access among racial and ethnic minorities, uncovering extensive inequalities, yet research into disparities in palliative care remains scarce within the broader healthcare sector (Adsersen et al. 2023; Johnson and Rhodes 2016; Roydhouse et al. 2023).

Despite the increasing numbers of migrants in many Western nations, many of whom are aging and dying in a globalized and multicultural context, research into their end-of-life preferences is limited (Gerber et al. 2020; McAuliffe and Triandafyllidou 2022). To mitigate these disparities and enhance culturally appropriate care, the reviewed studies suggested several recommendations. These include actively engaging with African migrant communities to understand their unique perspectives and preferences, advocating for policy changes to ensure equitable access to palliative and end-of-life care, and providing targeted training to enhance healthcare providers’ cultural competency and understanding of diverse needs (de Graaff et al. 2012; Hiruy and Mwanri 2014; Maddalena et al. 2010; Nyashanu et al. 2020). Additionally, conducting well-funded, targeted, and culturally sensitive research that considers cultural and religious preferences can guide stakeholders and enhance migrant and minority health, including end-of-life care (El Alaoui-Faris 2022; Hiruy and Mwanri 2014; Renzaho et al. 2016). The WHO has also advocated for primary research investments to disaggregate health data by subpopulation for equitable care (World Health Organisation 2018). Other studies suggest that research should differentiate migrant groups based on their unique religious, ethnic, or cultural backgrounds to avoid homogenizing them and generalizing their health status (Grisold and Grisold 2022; Venters and Gany 2011).

The review identified general limitations in participants’ awareness of Western end-of-life and palliative care advantages, which influenced their perceptions and experiences (de Graaff et al. 2012; Hiruy and Mwanri 2014; Maddalena et al. 2010). The reviewed studies stressed the importance of healthcare providers enhancing their cultural competencies by appreciating diverse experiences, accommodating differences, and aligning practices to meet the cultural needs and expectations of racial and ethnic communities. These studies also show that migrants are often unfamiliar with end-of-life planning processes common in Western societies, such as preparing advance care directives (de Graaff et al. 2012; Maddalena et al. 2010; Nyashanu et al. 2020; Sneesby et al. 2011).

By recognizing and addressing biases, understanding patients’ cultural backgrounds, and delivering respectful and responsive care, practitioners can bridge gaps in cultural understanding (Aldridge and Kutner 2014; Cherny 2009). Additionally, healthcare professionals can mitigate cultural misunderstandings and improve access and utilization of services for minority patients in palliative care settings through open dialogue about their varied healthcare needs and preferences (Gerber et al. 2020). Promoting inclusive healthcare environments can enhance cultural safety, foster cultural identity, and enrich end-of-life experiences for migrant and ethnic patients and their families, contributing to a more equitable care landscape. Although challenging in the short term, educating healthcare workers on ethnic health belief diversities, behaviors, and preferences is a step toward reducing disparities in health outcomes between migrants and native populations, ensuring culturally respectful and empathetic end-of-life care (Agyemang and Van Den Born 2019).

## Policy and procedure

The Australian *National Palliative Care Strategy 2018* provides a crucial framework for developing and implementing palliative care policies, strategies, and services in Australia (Department of Health and Aged Care 2019). However, while this policy initiative is broadly relevant to the review’s topic, its generalizability is limited due to the diverse social contexts and unique characteristics of these populations. Migrant populations exhibit significant heterogeneity, differing in legal status, cultural background, language, and socio-economic conditions (OECD 2019). This diversity substantially impacts their integration experiences and needs, underscoring the importance of considering this heterogeneity in policy-making and social integration efforts.

The reviewed studies proposed several policy strategies to address healthcare disparities and better support patients and families. These strategies include targeted education campaigns, improved language translation and interpretation services for non-English speaking migrants, and culturally sensitive care guidelines for healthcare practitioners (Ben-Arye et al. 2018; Coe 2020; de Graaff et al. 2012; Hiruy and Mwanri 2014; Maddalena et al. 2010; Nyashanu et al. 2020; Sneesby et al. 2011).

## Limitations and strengths

This scoping review encountered several methodological limitations. Despite employing a comprehensive search strategy, some relevant published and unpublished literature might have been missed. Additionally, limiting the inclusion to English-language studies potentially excluded important non-English research. The review was also constrained by the limited availability of grey literature specifically addressing African migrant populations and the phenomenon of interest.

However, this review also has notable strengths. Prioritizing peer-reviewed studies ensured the inclusion of high-quality research, which generally demonstrates higher validity compared to non-peer-reviewed sources (Conn et al. 2003). The exclusion of grey literature, due to concerns about methodological rigor and reliability, further enhanced the review’s findings (Shrivastava and Mahajan 2021). Moreover, requiring peer review and critical evaluation by field experts ensured adherence to standards of quality, validity, and reliability (Shamseer et al. 2015).

To ensure methodological rigor, we used a triangulated study selection and data extraction process. Two independent reviewers conducted these processes, with any discrepancies resolved by a third reviewer. Additionally, to enhance reliability, transparency, and validity, we documented post-protocol amendments, as recommended by Moher et al. (2009) and Tricco et al. (2018). Specifically, we revised the review’s title, research questions, and inclusion/exclusion criteria to incorporate the descriptive elements of the PCC mnemonic.

We also adopted a deductive data analysis method post-protocol, constructing a conceptual framework utilized to analyze the included studies and anticipate the presence of predetermined concepts (Azungah 2018). This review adhered to the PRISMA-ScR (Preferred Reporting Items for Systematic Reviews and Meta-analyses) extension for Scoping Review guidelines for transparent reporting (Tricco et al. 2018), ensuring a systematic and comprehensive examination of the existing literature.

## Conclusion

This review highlights a significant gap in understanding the palliative care needs of migrants, particularly within the African migrant cohort. Factors such as reluctance to discuss death, linguistic barriers, and varying health literacy levels may contribute to care delays and inequities. While conventional Western palliative care may suit most patients, it often clashes with the cultural and ethical expectations of migrant and ethnic minority groups (Grisold and Grisold 2022), including their preference for home-based care. Research also shows that racial and ethnic minorities disproportionately underutilize palliative care services, leading to prolonged aggressive therapies (Smith and Brawley 2014). Improving awareness and literacy may enhance acceptance of Western palliative care models. Additionally, enhancing cultural competency among healthcare practitioners is essential for equitable, culturally responsive healthcare.

African migrants remain underrepresented in health research, with their diverse ethnic heterogeneity, cultural, and religious practices often overlooked (Addo 2022; Arthur et al. 2012; Renzaho et al. 2016). To address this, focused, culturally sensitive research is needed to inform and maintain a sustained dedication to understanding the cultural determinants shaping health and illness perceptions and experiences (El Alaoui-Faris 2022), including in end-of-life stages.

## Appendix B: Search strategy and results for CINAHL database

**Table:**

Search ID #	Query	Limiters/expanders	Last run via	Results
S4	S1 AND S2 AND S3	Expanders – Apply equivalent subject sSearch modes – Proximity	Interface – EBSCOhost Research Databases Search Screen – Advanced Search Database – CINAHL Ultimate	11
S3	(African migrant* or African refugee* and (in Australia)) OR (African migrant* or refugee* and (in Australia)) OR African Australian	Expanders – Apply equivalent subjects Search modes – Proximity	Interface – EBSCOhost Research Databases Search Screen – Advanced Search Database – CINAHL Ultimate	925
S2	(Experiences OR perceptions OR views OR attitudes) OR (understanding OR knowledge or awareness OR beliefs OR values)	Expanders – Apply equivalent subjects Search modes – Proximity	Interface – EBSCOhost Research Databases Search Screen – Advanced Search Database – CINAHL Ultimate	1,860,840
S1	(End of life care OR palliative care OR hospice care) OR (death OR dying OR terminally ill) OR (end-of-life OR eol OR eolc)	Expanders – Apply equivalent subjects Search modes – Proximity	Interface – EBSCOhost Research Databases Search Screen – Advanced Search Database – CINAHL Ultimate	334,023
